# SOCIAL CONTEXTS AND WELL-BEING IN AGING: EXPLORING NEIGHBORHOOD ENVIRONMENT, NETWORKS, FAMILY DYNAMICS, AND LONELINESS

**DOI:** 10.1093/geroni/igae098.1532

**Published:** 2024-12-31

**Authors:** Hui Liu, Deborah Carr

**Affiliations:** Chair; Discussant

## Abstract

The five papers presented in this symposium utilize data from the National Social Life, Health, and Aging Project (NSHAP) to investigate the impact of social context, encompassing neighborhood environment, social networks, family dynamics, and social isolation, on health and well-being in later life. Ashtiani and Moorman examine the influence of neighborhood factors on loneliness, revealing that the residential environment indirectly affects loneliness through perceived social cohesion and neighborhood characteristics. Li, Wong, and Waite explore the long-term dyadic effects of adverse childhood experiences (ACEs) on later-life social participation, while considering the moderating role of spousal support and strain. Their findings demonstrate that both individual and spousal ACEs correlate with diminished social participation in later life, with spousal effects more pronounced from husbands to wives and further varied by marital quality. Choi investigates the association between marital history and the availability, quality, and stability of older adults’ social network ties to their children, uncovering significant impacts of past and present marital experiences on parent-child relationships. Additionally, two other papers employ comparative analyses, drawing from NSHAP and European datasets. Iveniuk, Schafer, and Sun assess the extent to which the link between education and cognition is mediated by social network characteristics, revealing variations in findings across the U.S. and European countries. Azar examines variations in the relationships among loneliness, social isolation, and health across different institutional exposures to defamilization policies in the U.S. and Europe over the life course.

